# Functional Characterization of a Lipoprotein-Encoding Operon in *Campylobacter jejuni*

**DOI:** 10.1371/journal.pone.0020084

**Published:** 2011-05-23

**Authors:** Mayumi Oakland, Byeonghwa Jeon, Orhan Sahin, Zhangqi Shen, Qijing Zhang

**Affiliations:** Department of Veterinary Microbiology and Preventive Medicine, Iowa State University, Ames, Iowa, United States of America; University of Osnabrueck, Germany

## Abstract

**Background:**

Bacterial lipoproteins have important functions in bacterial pathogenesis and physiology. In *Campylobacter jejuni*, a major foodborne pathogen causing gastroenteritis in humans, the majority of lipoproteins have not been functionally characterized. Previously, we showed by DNA microarray that CmeR, a transcriptional regulator repressing the expression of the multidrug efflux pump CmeABC, modulates the expression of a three-gene operon (*cj0089*, *cj0090*, and *cj0091*) encoding a cluster of lipoproteins in *C. jejuni*.

**Methodology/Principal Findings:**

In this work, we characterized the function and regulation of the *cj0089*-*cj0090*-*cj0091* operon. In contrast to the repression of *cmeABC*, CmeR activates the expression of the lipoprotein genes and the regulation is confirmed by immunoblotting using anti-Cj0089 and anti-Cj0091 antibodies. Gel mobility shift assay showed that CmeR directly binds to the promoter of the lipoprotein operon, but the binding is much weaker compared with the promoter of *cmeABC*. Analysis of different cellular fractions indicated that Cj0089 was associated with the inner membrane, while Cj0091 was located on the outer membrane. Inactivation of *cj0091*, but not *cj0089*, significantly reduced the adherence of *C. jejuni* to INT 407 cells *in vitro*, indicating that Cj0091 has a function in adherence. When inoculated into chickens, the Cj0091 mutant also showed a defect in early colonization of the intestinal tract, suggesting that Cj0091 contributes to *Campylobacter* colonization *in vivo*. It was also shown that Cj0091 was produced and immunogenic in chickens that were naturally infected by *C. jejuni*.

**Conclusion/Significance:**

These results indicate that the lipoprotein operon is subject to direct regulation by CmeR and that Cj0091 functions as an adhesion mechanism in *C. jejuni* and contributes to *Campylobacter* colonization of the intestinal tract in animal hosts.

## Introduction

*Campylobacter jejuni* is a Gram-negative, curved or spirally shaped bacterium with a single, polar, unsheathed flagellum at one or both ends [1]. It is a commensal organism existing in the intestinal tracts of a variety of wild and domestic animals, especially in birds. *C. jejuni* is a leading cause of acute diarrhea in humans worldwide [2]. The typical symptoms of *Campylobacter* infections in humans include watery to bloody diarrhea, abdominal pain, fever, and presence of leukocytes and red blood cells in feces [3]. *Campylobacter* infections can also develop to Guillain-Barré syndrome (GBS), an autoimmune-mediated neurodegenerative disorder which causes acute neuromuscular paralysis [4]. The most significant source of *Campylobacter* infections for humans is the consumption of undercooked chicken.

The pathogenic process of *C. jejuni* in humans has not been well understood, but can be divided into several stages [5]. Once ingested by the host, *C. jejuni* survives the stresses in the stomach and small intestine. Upon reaching to the large intestinal tract, *C. jejuni* colonizes mucus layer and adheres to the intestinal cell surface of the host gastrointestinal tract. The organism produces a cytolethal distending toxin (CDT) and possibly other toxins, but their role in pathogenesis is not clear [6]. Once adhered to the host intestinal epithelial cells, *C. jejuni* may invade into and proliferate within the host cells. The invasion and proliferation of the organism inside host cells are considered the cause of cell damage and induce host inflammatory responses, which result in diarrhea with fecal leukocytes [7]. Occasionally *C. jejuni* can spread to extraintestinal sites, such as liver, gallbladder, pancreas, uterus, and fetal tissues [3], [7].

The known putative virulence factors involved in *Campylobacter* pathogenesis include flagella, lipooligosaccharide (LOS), CDT, and outer membrane proteins [7]. Flagella aid *Campylobacter* to move through the mucus layer and contribute to colonization and invasion [8]. LOS is involved in adherence to host cells and serves as an endotoxin that induces host intestinal inflammatory responses [7]. In addition, molecular mimicry of LOS to human gangliosides is considered a key factor in the development of GBS [9]. CDT causes cell cycle arrest and host DNA damage, which induce host inflammatory responses [10]. The outer membrane proteins of *Campylobacter* are involved in interactions with hosts and play important roles in adherence and colonization. CadF, a 37-kDa surface protein, binds to fibronectins located at cell-to-cell contact regions in the gastrointestinal epithelium. CadF is required for *Campylobacter* colonization of chickens [11], [12]. PEB1 is a periplasmic protein homologous to a solute-binding component of amino acid ABC transporters [13]. PEB1 is important for *C. jejuni* adherence to human cells and colonization in the intestinal tract of mice [14]. The major outer membrane protein (MOMP), a 45-kDa porin, adheres *in vitro* to human intestinal cell membranes and fibronectin [15], but whether it is involved in *in vivo* adherence is unknown. CmeABC functions as an efflux pump to extrude a variety of substrates such as antibiotics, ions, SDS, and bile salts [16]–[18]. In addition, CmeABC mediates bile resistance and is required for *C. jejuni* colonization in the gastrointestinal tract of chickens [16].

Bacterial lipoproteins have diverse functions including cell adhesion, transport, nutrient acquisition, mating, and serum resistance as well as stimulation of inflammatory/immune responses in host cells [19]. *C. jejuni* has multiple membrane lipoproteins predicted from the genomic sequences [19]. At present, only four of these lipoproteins, JlpA [20] and CapA [21], CjaA [22], and FlpA [23] have been functionally characterized in *C. jejuni*. JlpA, a surface-exposed, loosely cell-associated lipoprotein, is involved in the interaction of *C. jejuni* with the surface-exposed heat shock protein 90α (Hsp90α) of host cells and triggers signal transduction, leading to the activation of components (NF-κB and p38 MAP kinase) involved in host proinflammatory responses to infections [24]. CapA is also involved in *Campylobacter* adherence to host epithelial cells and colonization in gastrointestinal tract of chicken [21]. CjaA is an inner-membrane associated lipoprotein, and has been shown that immunization of chickens with avirulent *Salmonella* strain expressing CjaA reduced the colonization of the intestinal tract by *C. jejuni* [22]. FlpA is a putative outer membrane-associated lipoprotein, which mediates adherence to chicken and human epithelial cells as well as chicken colonization via binding to fibronectin [23], [25].

In a previous study comparing the global gene expression profiles of *in vitro* grown NCTC 11168 and its isogenic CmeR mutant using DNA microarray [26], we found that CmeR, which is a transcriptional repressor for the multidrug efflux pump CmeABC [27], functions as a pleiotropic regulator modulating the expression of multiple genes in *C. jejuni* NCTC 11168 [26]. In total, 28 genes showed ≥2-fold changes in expression in the CmeR-deletion mutant compared with the wild-type strain. Among the CmeR-regulated targets were *cj0089 and cj0091* encoding putative lipoproteins. *cj0089* and *cj0091*, along with *cj0090* (also encoding a putative lipoprotein) appear to be organized into an operon, but their detailed regulatory mechanisms and the role in *Campylobacter* pathophysiology remain unknown. Considering the fact that bacterial lipoproteins have important functions and the majority of lipoproteins in *C. jejuni* have not been characterized, we conducted both *in vitro* and *in vivo* experiments to elucidate the regulation of the lipoprotein-encoding operon and the functions of the encoded products in *Campylobacter* adherence and colonization.

## Results

### Genomic organization and co-transcription of *cj0089, cj0090,* and *cj0091*

**Figure 1:**
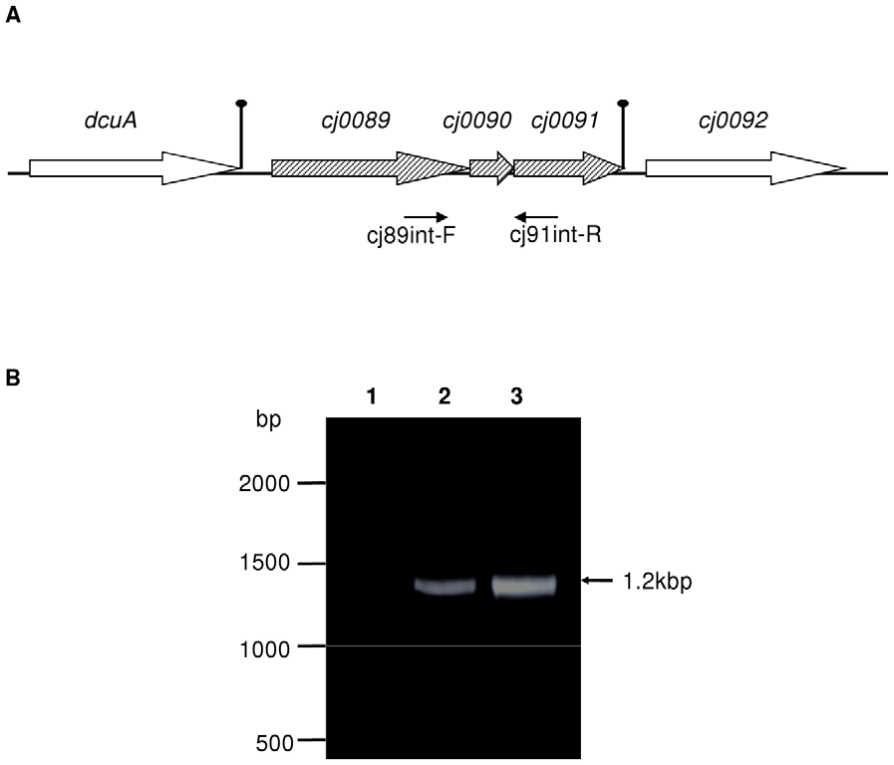
Genomic organization and co-transcription of *cj0089*, *cj0090*, and *cj0091*. (A) The genes are depicted by boxed arrows. The locations of the primers used for RT-PCR were indicated by arrows. Predicted stem-loop encoding regions are shown by vertical oval arrows. (B) RT-PCR was performed to determine the co-transcription of *cj0089*, *cj0090*, and *cj0091* using primers, cj89int-F and cj91int-R. Lane 1, no RT control; lane 2, RNA with RT and DNA polymerase; lane 3, Positive control (DNA was used as the template).

The three genes encoding the cluster of lipoproteins are tandemly positioned in the chromosome of *C. jejuni* NCTC 11168 (Figure 1A). *cj0089* and *cj0090* are separated by 9 nucleotides, while *cj0090* and *cj0091* are separated by 23 nucleotides. No predicted stem-loop structures exist between the ORFs. According to the prediction by Petersen *et al*. [28], there is a putative RpoD promoter located upstream of *cj0089*. The TATA-box of this promoter is located 40 nucleotides upstream of the *cj0089* translational initiation codon (data not shown). No promoter was predicted immediately upstream of *cj0090* or cj0091. To determine whether cj0089, cj0090, and cj0091 are co-transcribed, RT-PCR was performed on the C. jejuni strain using primers cj89int-F and cj91int-R (Tables 1 and 2), which span the three ORFs (Figure 1A). A 1194-bp expected product was amplified in the PCR reaction with the RNA and RT, while no product was obtained in the reaction without RT, indicating that *cj0089*, *cj0090*, and *cj0091* are con-transcribed and likely organized into an operon (Figure 1B). The operon is flanked by an upstream gene encoding an anaerobic C4-dicarboxylate transporter (*dcuA*) and a downstream gene (*cj0092*) encoding a hypothetical protein. There is a potential Rho-independent transcriptional terminator forming a 23-nucleotide stem-loop structure located immediately downstream of *dcuA* (Figure 1A). Another stem-loop structure is present downstream of *cj0091* (Figure 1A). These sequence features further support the notion that the three lipoprotein genes form an operon.

**Table 1:** Bacterial strains and plasmids used in this study.

Plasmid or strain	Relevant characteristics	Source
Strains		
*C. jejuni*		
NCTC 11168	Wild type	[57]
Cj89-	11168 derivative, *cj0089::Kan^r^*	This study
Cj91-	11168 derivative, *cj0091::Kan^ract^*	This study
Comp91	Cj91- complemented with pUOA18-Comp91	This study
*E. coli*		
DH5α	F-Φ80lacZΔM15Δ(lacZYA-argF)U169recA1endA1hsdR17*(r_k_* ^−^,*m_k_^+^)phoAsupE44thi-1gyrA96relA1λ* ^−^	Invitrogen
JN109	endA1 recA gyrA96 thi hsdR17*(r_k_* ^−^,*m_k_^+^) relA1supE44Δ(lac-proAB)[FreaD36proABlacI^q^ZΔM15]*	Promega
Plasmids		
pUC19	*E. coli* cloning vector	[58]
pUC19-89	pUC19 containing full-length *cj0089, Amp^r^*	This study
pUC19-89K	pUC19-89 derivative carrying *cj0089::Kan^r^*	This study
pUC19-91	pUC19 containing full-length *cj0091, Amp^r^*	This study
pUC19-91K	pUC19-89 derivative carrying *cj0091::Kan^r^*	This study
pRY112	*E. coli-C. jejuni* shuttle vector, *Cm^r^*	[52]
pRY112-Comp91	pRY112 containing containing the promoter region of *cmeABC* and full-length Cj0091	This study
pUOA18	*E. coli-C. jejuni* shuttle vector, *Cm^r^*	[53]
pUOA18-Comp91	pUOA18 containing containing the promoter region of *cmeABC* and full-length Cj0091	This study
pMW10	*E. coli-C. jejuni* shuttle vector with promoterless E. *coli lacZ* gene, *Kan^r^*	[51]

**Table 2:** Primer sequences used in this study.

Primers	Sequences
cj0089-F5	5′-GAGgcatgcCCGACTTTGTTAGGTGCAGTGCAA-3′ (*Sph*I)
cj0089-R5	5′-GCAgagctcGCTGGAATTCTAAGGGCTTGATAGTCCT-3′ (*Sac*I)
cj89-U2	5′-GCTccatggTGCCTTATCTTGACGCATTAAAACACG-3′ (*Nco*I)
cj89-L2	5′-CAGccatggGGGAAGCTTCTTTTGCAAATTTAAATATAAATGG-3′ (*Nco*I)
cj0091-F2	5′-GTGCGTtctagaAGAGGTTTACCAAAAAGTA-3′ (*Xba*I)
cj0091-R2	5′-CTTTTGGgagctcATCAAATTCCTTAGCATTCGTA-3′ (*Sac*I)
cj0089F	5′-AGTggatccTGCCTTTTTTTAACAGCTTGTG-3′ (*BamH*I)
cj0089R	5′-GAGgagctcTTATTTTTCCATGATAGCAAC-3′ *(Sac*I)
cj0091-F1	5′-AGTggatccTGTGCGCAAACAGCTTATACAGATGGAAAG-3′ (*BamH*I)
cj0091-R1	5′-GAGgagctcTTACCAAGTAACTGATTTACTAGAACCGGTTTTATC-3′(*Sac*I)
Pcj89-F1	5′-AGGCTTTGTATTAGCTCCTATGCTTAT-3′
Pcj89-R1	5′-CAAGCAATACCTGAAAAAATCAACCCAA-3′
Cj89int-F	5′-TTTAAAATAAATATGCCTGCAATGA-3′
Cj89int-R	5′-GCGATACCAATGTCCCTTTTT-3′
KanNco-F	5′-CTTATCAATATATccatggAATGGGCAAAGCAT-3′(*Nco*I)
KanNco-R	5′-GATAGAAccatggATAATGCTAAGACAATCACTAAA-3′(*Nco*I)
Comp91-F2	5′-CAAccgcggATGATTTTAGATTAGAAATTAAAGCAA-3′(*Sac*I)
Comp91-R2	5′-GCCCgagctcAACACTTGAGCTAAAA-3′(*Sac*II)
Comp91-F3	5′-GCTtctagaTGGAATCAATAGCTCCAAAGCTTAA-3′(*Xba*I)

*The restriction sites in the sequences are indicated by lower cases. The corresponding restriction enzymes for the restriction sites were indicated in parenthesis.

### Predicted features of the putative lipoproteins

Analysis of the predicted amino acid sequences indicated that *cj0089*, *cj0090*, and *cj0091* encode putative lipoproteins because each of them has a typical N-terminal lipoprotein signal peptide. LipoP algorithm [29] predicted a signal peptidase II cleavage site at LFLTA↓C for Cj0089, at FLLSA↓C for Cj0090, and at LLFSG↓C for Cj0091. Cj0089 (453 amino acids), Cj0090 (122 amino acids), and Cj0091 (207 amino acids) each has a calculated molecular mass of 51.28 kDa, 13.92 kDa, and 22.32 kDa, respectively. The molecular masses of the mature lipoproteins, after cleavage of the signal peptides, are predicted to be 49.4 kDa, 12.29 kDa, and 20.51 kDa, respectively. BLASTP searches against the public non-redundant protein database (http://www.ncbi.nlm.nih.gov/) showed that Cj0089 bears similarity (78–89%) to tetratricopeptide TPR-2 repeat proteins found in *Shewanella* spp. The functions of these tetratricopeptide TPR-2 repeat proteins have not been characterized. Cj0089 also has a 27.4% identity to HP0018, a hypothetical protein in *Helicobacter pylori*. Cj0091 is 33.5% identical to HP1457 (hypothetical protein) and has a conserved domain similar to the collagen-binding surface adhesin, SpaP (antigen I/II family) of *Yersinia pestis* biovar Orientalis [30], where its function has not been determined empirically. Cj0090 has a 46% identity to a *Helicobacter pylori* protein (HP0444; hypothetical protein). In this study, we choose to focus on characterization of Cj0089 and Cj0091.

### Production of rCj0089 and rCj0091 and their specific antibodies

The coding sequences of *cj0089* and *cj0091*, excluding their signal peptides, were separately cloned into pQE-30 vector, expressed in *E. coli* JM109, and the proteins purified by affinity chromatography. Using the purified proteins, rabbit polyclonal antibodies against rCj0089 and rCj0091 were produced. The anti-Cj0089 antibody recognized a protein with a molecular mass of 49 kDa in NCTC 11168. On the other hand, anti-Cj0091 recognized a protein with a molecular mass of approximately 18 kDa, slightly smaller than the calculated mass of Cj0091 (20.51 kDa) (data not shown). These immunoblotting results indicated that both lipoproteins were produced in *C. jejuni* when grown in MH broth.

### Regulation of *cj0089* and *cj0091* by CmeR

**Figure 2:**
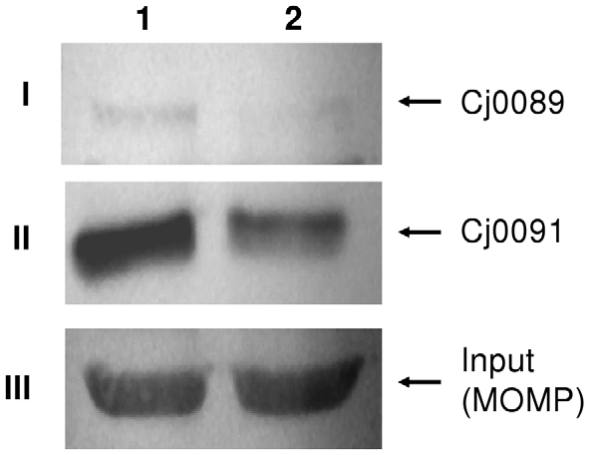
Effect of c*meR* mutation on the expression of Cj0089 and Cj0091 as determined by immunoblotting. Whole cell lysates of strains NCTC 11168 (lane 1) and the *cmeR* mutant (lane 2) were separated by SDS-PAGE and probed with anti-rCj0089 (panel I), anti-rCj0091 (panel II), and anti-rMOMP (panel III). MOMP, a 45-kDa outer membrane protein, was used as an internal control for protein loading.

In a previous DNA microarray study by our research group [26], it was found that *cj0089* and *cj0091* were down-regulated in the *cmeR* mutant, suggesting that CmeR activates the expression of the lipoprotein genes. To confirm the previous finding at the protein level, the expression levels of Cj0089 and Cj0091 in the wild-type strain and in the *cmeR* mutant were analyzed by immunoblotting using anti-rCj0089 and anti-rCj0091 antibodies. The same amount of total proteins was loaded in each lane and additionally MOMP (a major outer membrane protein present in all *C. jejuni* isolates) was used as in internal control for protein loading in each lane. The immuoblotting results clearly showed that the production of Cj0089 and Cj0091 in the *cmeR* mutant was decreased compared with that in the wild-type (Figure 2). According to the densitometric analysis of immunoblots using AlphaEase® software (Alpha Innotech), the band intensity of Cj0089 was 2.98 fold higher in the wild-type than that in the c*meR* mutant, and the band intensity of Cj0091 was 1.92 fold higher in the wild-type than that in the *cmeR* mutant. The immunoblotting data further confirmed the results of the DNA microarray and real-time RT-PCR [26].

**Figure 3:**
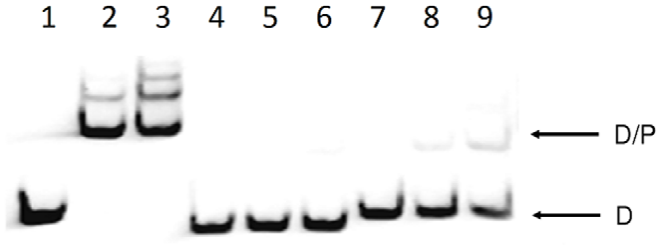
Binding of rCmeR to the promoter DNA of the lipoprotein operon as determined by EMSA. The positive control DNA (promoter of *cmeABC*; lanes 1 to 3), negative control DNA (coding sequence of *cmeB;* lanes 4 to 6), and the promoter DNA of the lipoprotein operon (lanes 7 to 9) are included in the same gel. In the reactions, a fixed amount of DIG-labeled DNA (0.02 pmol) was incubated with 0 (lanes 1, 4, and 7), 20 (lanes 2, 5, and 8), or 40 ng (lanes 3, 6, and 9) of rCmeR, respectively. The position of the DIG-labeled DNA (D) and DNA-rCmeR complex (D/P) are indicated.

To determine if CmeR directly or indirectly regulates the lipoprotein operon, a gel mobility shift assay (EMSA) was performed using rCmeR and the promoter sequence upstream of *cj0089*. The promoter DNA was amplified with primers Pcj89-F1 and Pcj89-R1 (Table 2). rCmeR bound to the promoter region of the lipoprotein operon (Figure 3). However, the binding was significantly weaker compared with that of the *cmeABC* promoter, suggesting that CmeR has a low affinity to the lipoprotein promoter. Regardless, the finding from EMSA suggests that this lipoprotein operon is subject to direct regulation by CmeR.

### Cj0089 and Cj0091 are membrane-associated proteins

**Figure 4:**
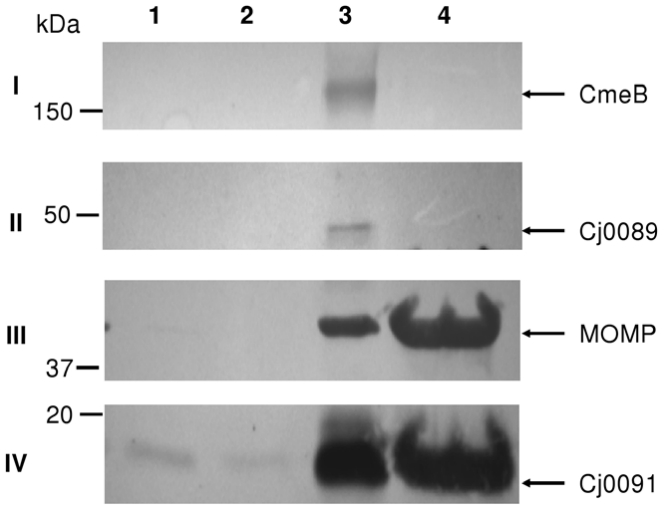
Localization of Cj0089 and Cj0091 in *C. jejuni*. The cytosol fraction (lane 1), periplasmic fraction (lane 2), inner membrane fraction (lane 3), and outer membrane fraction (lane 4) were separated by SDS-PAGE and probed with anti-rCmeB (panel I), anti-rCj0089 (panel II), anti-rMOMP (panel III), and anti-rCj0091 (panel IV), respectively.

In order to determine the cellular localization of Cj0089 and Cj0091, *C. jejuni* strain NCTC 11168 was fractionated to isolate proteins from the cytosol, periplasm, inner membrane, and outer membrane. The fractions were examined by immunoblotting with anti-rCj0089 and anti-rCj0091 antibodies (Figure 4). Cj0089 was detected predominantly in the inner membrane fraction while Cj0091 was found in both the inner and outer membrane fractions, but the majority of Cj0091 was in the outer membrane. Antisera against rCmeB, an inner membrane drug transporter of multidrug efflux pump CmeABC [16], and against the recombinant major outer membrane protein (MOMP) [31], were used as controls for the membrane fractions. CmeB is exclusively detected in the inner membrane fraction; however, MOMP was detected in both outer membrane and inner membrane fractions, with the majority of MOMP was being in the outer membrane fraction (Figure 4). The results from the controls suggested that the inner membrane fraction contained some outer membrane proteins, while the outer membrane fraction was pure. Based on the blotting results and in reference to the controls, it was concluded that Cj0089 is associated with the inner membrane, while Cj0091 is associated with outer membrane of *C. jejuni*.

### Cj0089 and Cj0091 were inactivated in the insertional mutants

**Figure 5:**
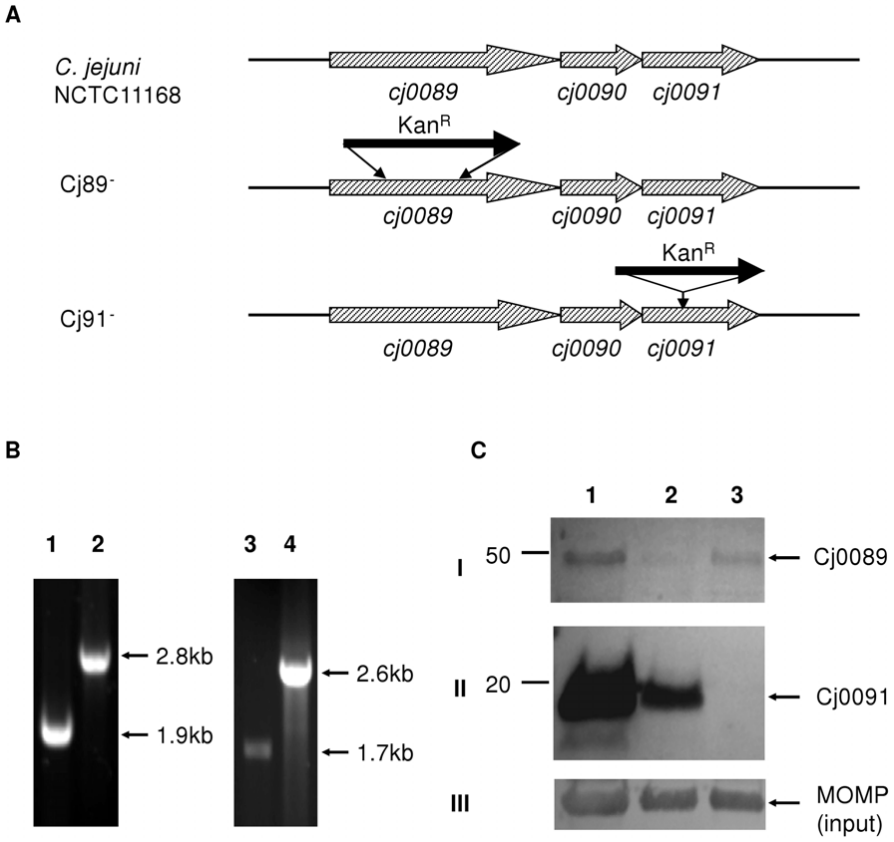
Generation of deletional and insertional mutations in *cj0089* and *cj0091*. (A) Schematic diagram of the locations of deletional and insertional mutations. The genes are indicated by boxed arrows. The locations of the Kan^R^ insertion are indicated as solid arrows. (B) PCR confirmation of the insertions in Cj89^−^ and Cj91^−^. Lanes 1, *cj0089* in wild-type strain; lane 2, *cj0089* in Cj89^−^; lane 3, *cj0091* in wild-type strain; and lane 4, *cj0091* in cj91^−^. (C) Immunoblotting analysis of protein production in various constructs. Wild-type (lane 1), Cj89^−^(lane 2), and Cj91^−^(lane 3) were separated by SDS-PAGE and probed with anti-rCj0089 (panel I), anti-rCj0091 (panel II), and anti-rMOMP (panel III). MOMP, a 45-kDa outer membrane protein, was used as a loading control.

To study the functions of the lipoproteins, insertional mutants of *cj0089* and *cj0091* were generated (Figure 5A). Correct insertion and orientation of the Kan^R^ cassette in Cj89^−^ and Cj91^−^ were confirmed by PCR (Figure 5B). The disruptions of *cj0089* and *cj0091* were also confirmed by immunoblotting using anti-rCj0089 and anti-rCj0091 (Figure 5C). In Cj89^−^, the expression level of *cj0091* was significantly decreased, but was not abolished (Figure 5C, panel II, lane 2), indicating that the insertion in *cj0089* caused a partial polar effect on *cj0091*.

### Cj0091 is required for *Campylobacter* adherence to INT 407 cells *in vitro*

**Figure 6:**
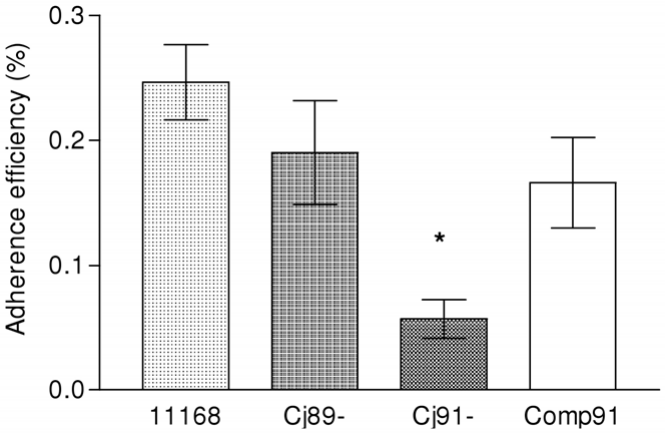
Adherence of *C. jejuni* strain NCTC 11168 and the mutants to INT 407 cells. Strain NCTC 11168, Cj89^−^, and Cj91^−^ were inoculated onto monolayers of INT 407 cells and the percentages of adherent bacterial cells were calculated by viable plate counts. Each experiment was conducted in quadruplicate and repeated for 3 times. Bars and error bars indicate the mean ± standard deviation. * indicates significant differences in colonization levels at P<0.05 level as determined by Student's t-test.

Since the results indicated that Cj0091 is an outer membrane-associated protein and has a domain homologous to SpaP involved in surface adherence, it is possible that Cj0091 is involved in the interaction between *C. jejuni* and host cells. To examine this possibility, the ability of NCTC 11168 wild type strain and the two isogenic mutants, Cj89^−^ and Cj91^−^, to adhere to the monolayers of human intestinal cell line INT 407 was measured (Figure 6). Cj91^−^ showed a 4.3-fold reduction in adherence compared with the wild-type strain (P<0.05). Cj89^−^ had a 1.3-fold reduction in adherence compared to the wild-type; however, the difference was not significant (p>0.05). The adherence ability of the complemented strain of Cj91^−^, Comp91, to INT 407 cell line was restored partially to the wild-type level (Figure 6). To rule out the possibility that the observed phenotype of Cj91^−^ was due to its increased sensitivity to Triton X-100 (used in the adhesion assay to dissociate bacteria from the INT 407 cells), both the wild-type and Cj91^−^strains were treated with 1% Triton X-100 for 20 min and then the treated bacteria were plated onto MH agar plates for CFU counts. The result indicated that both strains were equally resistant to the treatment with Triton X-100 (result not shown). These findings indicate that Cj0091, but not Cj0089 has a significant role in *Campylobacter* adherence to INT 407 cells.

### Cj0091 is involved in intestinal colonization in chicken

**Figure 7:**
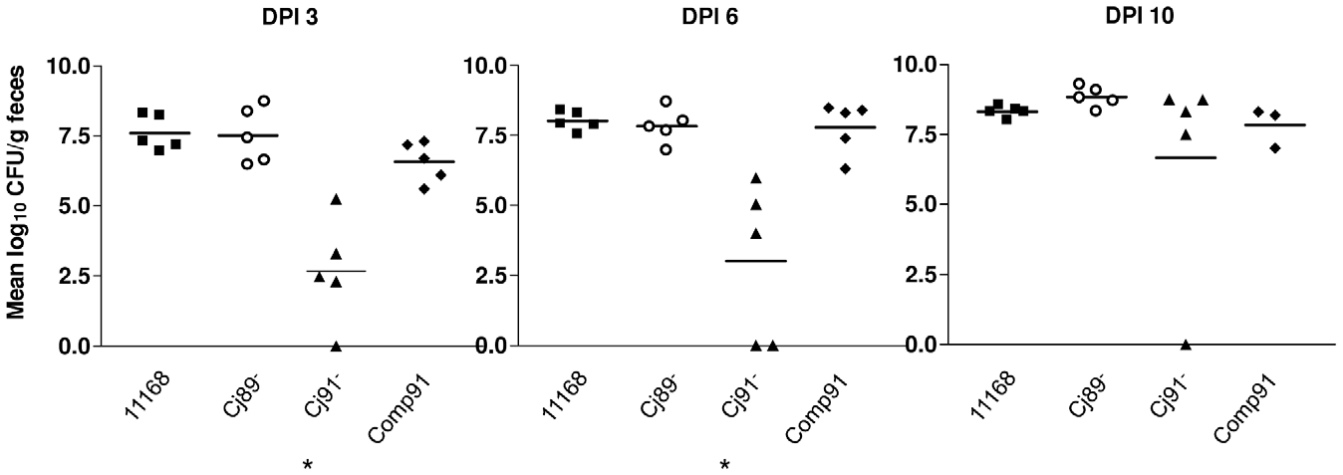
Colonization of *C. jejuni* strain NCTC 11168 and its mutants in chickens. Approximately 10^7^ CFU of each of NCTC 11168 (solid squares), Cj89^−^(open circles), Cj91^−^ (solid triangles), Comp91 (solid diamonds) were inoculated into 3-day-old chickens. Cecal contents were collected on 3, 6, and 10 days post inoculation (DPI) and enumerated by plate counts. Each symbol represents the colonization level in a single bird. The mean for each group is depicted by a horizontal bar. The detection limit of the plating method is about 100 CFU/g of feces. An asterisk indicates significant differences in colonization levels at P<0.05 as determined by Student's t-test.

To test whether Cj0089 and Cj0091 had a role in chicken colonization, the wild-type strain 11168, its isogenic mutants Cj89^−^ and Cj91^−^, and Comp91 were separately inoculated into four groups of chickens. At necropsy, cecal contents were collected on DPI 3, 6, and 9 from 5 chickens from each group and cultured for enumeration of *Campylobacter* using selective plate counting. On DPI 3 and 6, the mean level of colonization by Cj91^−^ was substantially lower (reduced approximately 2.8 and 2.7 log_10_ units, respectively) than that of the wild-type strain (Figure 7). The differences were statistically significant (p<0.05). However, on DPI 10 no significant differences in colonization between the wild-type and Cj91^−^ were observed. The level of colonization was restored to the wild-type level in the complemented strain, Comp91 (Figure 7). Throughout the experiment, no differences in colonization were observed between the wild-type and Cj89^−^ (Figure 7). The colonization reduction seen with Cj91^−^ was not attributable to the difference in *in vitro* growth rates or motility because growth and motility of the mutants were equally comparable to those of the wild-type strain (data not shown). Together, these results indicate that Cj0091, but not Cj0089, contributes to the establishment of colonization during the early stages of *C. jejuni* infection in the intestinal tract of chickens.

### Cj0091 is expressed and immunogenic in chickens

**Figure 8:**
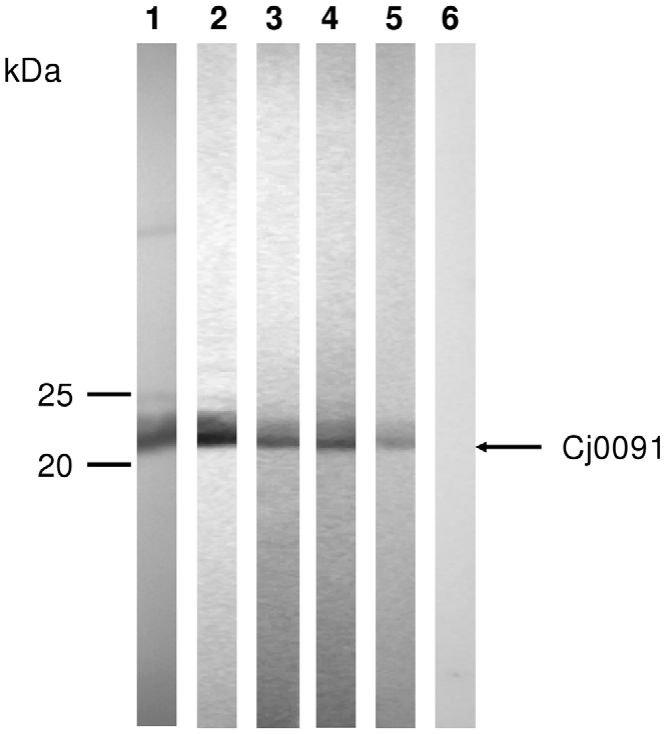
Immunoblotting detection of natural anti-Cj0091 antibodies in chickens. Strips of nitrocellulose membranes with rCj0091 were reacted with 4 chicken sera (lanes 2–5) that contained *Campylobacter*-specific maternal antibodies derived from natural infections and one chicken serum (lane 6) that was negative for anti-*Campylobacter* antibodies. Anti-rCj0091 was applied as a positive control (lane 1).

The contribution of Cj0091 to adherence and *in vivo* colonization prompted us to determine if Cj0091 was produced and immunogenic in chickens naturally infected by *Campylobacter*. For this purpose, rCj0091 was used as antigen in immunoblotting and was probed with chicken sera collected from 5 individual chickens, among which 4 contained natural maternal antibodies against *C. jejuni* and the other one was a negative control (*Campylobacter*-free and no anti-*Campylobacter* maternal antibodies) [32]. As shown in Figure 8, the 21-kDa rCj0091 was detected by the 4 sera, in which high level of *C. jejuni*-specific maternal antibodies (IgG) were present, but not by the serum in which the *C. jejuni*-specific maternal antibodies were absent (Figure 8). These findings strongly suggest that Cj0091 was produced and immunogenic during the colonization of chickens by *C. jejuni*.

## Discussion

In this study, we characterized an operon encoding three lipoproteins, Cj0089, Cj0090, and Cj0091. It was confirmed that this operon is activated and directly regulated by CmeR, a pleiotropic regulator modulating the expression of multiple genes with diverse functions in *Campylobacter* [26]. We also demonstrated that Cj0091, but not Cj0089, plays an important role in mediating *Campylobacter* adherence to INT 407 cells and in *Campylobacter* colonization of the gastrointestinal tract of chickens, especially in the early stage of the infection. These findings identify a new adhesion mechanism in *C. jejuni* and provide new insights into the pathogenesis of *C. jejuni*.

Homology searches using BLASTP showed that Cj0089 is similar to the tetratricopeptide TPR-2 repeat protein of *Shewanella* spp. (78–89% homology; E-value = 4e-19 to 4e-16). The tetratricopeptide repeat (TPR) is a structural motif, which mediates protein-protein interactions. TPR-containing proteins have diverse functions such as regulation of the cell cycle, protein transport, the regulation of transcription, splicing events, and protein folding [33], [34]. Proteins containing the TPR motif have been found in various organisms including bacteria, yeast, plants, insects, and animals [33], [34]. At present the function of Cj0089 is unknown, and the function of Cj0090 has yet to be determined. Since *cj0089*, *cj0090*, and *cj0091* form an operon, it is possible that the three encoded products have a functional link. This possibility remains to be defined in future studies. Cj0091 has a conserved domain homologous to the collagen-binding surface adhesin, SpaP (antigen I/II family) of *Y. pestis* biovar Orientalis. *spaP* was first identified in *Streptococcus mutans* as a gene encoding antigen I/II [35] and is also known as P1 [36]. The antigen P1, encoded by *spaP,* plays a role in adherence of *S. mutans* to the salivary pellicle of tooth surfaces or to other microorganisms [37], [38]. At this stage, it is unknown if Cj0091 binds to collagen or has functions similar to those of SpaP in *S. mutant*. It should be mentioned that Cj0089 and Cj0091 are putative lipoproteins as supported by bioinformatics analyses and membrane association experiments, and further studies such as palmitoylation assay using [^3^H]-palmitate are needed to prove that they are truly modified by lipids.

In this study we confirmed the regulation of the putative lipoprotein operon by CmeR (Figures 2 and 3). Based on the result of EMSA, CmeR directly interacts with the promoter DNA (Figure 3). However, the interaction is much weaker compared with the *cmeABC* promoter, suggesting that CmeR is a secondary regulator of the lipoprotein operon. This notion is supported by the fact that the fold change in expression of the lipoprotein genes was moderate as determined by microarray, real-time RT PCR, and immunoblotting. Analysis of the upstream sequence of *cj0089* did not identify the typical inverted repeat found in the promoter region of *cmeABC* recognized by CmeR [27]. Since CmeR serves as an activator for the lipoprotein operon, which is in contrast to the regulation of *cmeABC* (repressed by CmeR), the binding site for CmeR in front of the lipoprotein operon could be different from the one upstream *cmeABC*. This possibility awaits further inverstigation.

The contribution of Cj0091 to *Campylobacter* adherence was investigated using the human intestinal epithelial cell line INT 407 (Figure 6). So far, adhesins characterized in *C. jejuni* include PEB1 [14], CadF [11], MOMP [15], JlpA [20], CapA [21], FlpA [23], and Cj1349c [23]. PEB1 is a periplasmic protein, homologous to the periplasmic solute-binding protein component of amino acid ABC transporters [13]. An important function of PEB1 is that it binds to aspartate and glutamate, which are important sources of carbon and energy for *Campylobacter* [13], [39]. CadF, a surface protein, is involved in the binding of *C. jejuni* to fibronectin in the host gastrointestinal epithelium and stimulation of the host cell signal transduction pathway [11], [40]. MOMP, a trimeric outer membrane protein [41], [42], is involved in pore-forming activity [43], and assists outer membrane structural organization and stabilization [44]. Although one study suggested MOMP could be involved in adherence [15], the exact role of MOMP in the interaction between *Campylobacter* and host cells is unknown. JlpA is a lipoprotein and is involved in interaction with host cells *in vitro* [20]. Besides functioning as an adhesin, JlpA interacts with surface-exposed heat shock protein 90α (Hsp90α) on host cells, triggering signaling pathways and leading to the activation of NF-κB and p38 MAP kinase [24]. CapA, which is an autotransporter, is involved in the colonization of the chicken gut and plays a role in adherence to human epithelial cells [21]. FlpA contains Fn type III domains, and is involved in interaction with host cells *in vitro* and *in vivo* via binding to fibronectin [23], [25]. Cj1349c has been shown to be involved in adherence to chicken liver epithelial cells [23]. Thus, *C. jejuni* appears to possess multiple adhesins and the partial reduction of adherence seen with Cj91^−^ may be explained by the complementary effects of other adhesins.

Although we showed that Cj0091 is an outer membrane associated protein (Figure 4), it has yet to be determined whether this protein is located on the outer surface or is facing the periplasmic space. Controlled protease treatments did not yield conclusive result on the surface exposure of Cj0091 (data not shown). Therefore, it is uncertain how Cj0091 contributes to *Campylobacter* adherence to human epithelial cells. More advanced technologies (such as immunogold labeling and electron microscopy) may be used in future work to ascertain the exact location of Cj0091 on the outer membrane. It should be pointed out that not all adhesins are true outer surface exposed proteins. For example, PEB1 and CjaA are reported adhesins of *Campylobacter* and are mainly localized in the periplasmic space or inner membrane, but these proteins are shown to be secreted or attached on the outer surface, functioning as adhesins [22], [55]. Thus, even if Cj0091 faces inward on the outer membrane, this would still not exclude its role as an adhesin.

*C. jejuni* mainly colonizes in cecal and cloacal crypts in chickens [45], [46]. Unlike the colonization in mammals, such as mice, swine, rabbits, monkeys, and humans, where the organism commonly invades the host intestinal epithelial cells, *C. jejuni* does not usually invade the intestinal epithelium of chickens [47]–[49]. Thus, surface colonization is an important feature of *Campylobacter* infection in chickens. Ceca of chickens are blind-ended sacs filled with small food particles, fluid, and microorganisms. Cecal contents are constantly moving and periodically evacuated [50]. Thus, cecum is not a stagnant environment and *Campylobacter* must possess mechanisms for persistent colonization in this niche. Interestingly, the differences in colonization between the wild-type and Cj91^−^ were only seen at the early stage (before DPI 10) of infection (Figure 7). We speculate that this may reflect the possibility that Cj0091 is only required for the initial adherence and optimal establishment of infection, or that adaptation of the Cj91^−^ mutant occurs in the intestinal tract, overcoming the early defect in colonization. Although it has been reported that *Campylobacter* does not directly interact with intestinal epithelial cells, but rather resides in the mucus layer of the crypts moving freely and rapidly in chickens [46], it is highly likely that adherence to the gastrointestinal mucosal cell surface is still required for successful colonization in chickens [7]. Indeed, independent studies have shown that CapA, CadF, PEB1, and FlpA are each required for *Campylobacter* colonization in chickens [12], [14], [21], [23].

It was demonstrated in this study that Cj0089 is an inner membrane protein (Figure 4). Disruption of *cj0089* did not result in significant changes in *in vitro* adherence and *in vivo* colonization (Figures 6 and 7), suggesting that Cj0089 is not essential for *Campylobacter* growth in the intestinal tract. Alternatively, it may suggest that Cj0089 shares redundant function(s) with other genes in *C. jejuni*. Although *cj0089* and *cj0091* are located in the same operon and both are regulated by CmeR, they may have different functions in *Campylobacter* biology. Interestingly, mutation of *cj0089* reduced, but did not abolish, the expression of *cj0091* due to a polar effect (Figure 5). The partial reduction in Cj0091 expression did not cause an apparent phenotypic change, suggesting that partial production of Cj0091 is sufficient for adherence and colonization at the wild-type level.

Another interesting finding in this study is that Cj0091 is abundant in *C. jejuni in vitro* and is apparently immunogenic in chickens (Figure 8). To date, no commercial vaccines against *Campylobacter* are available for use in humans or poultry, but some promising candidates have been evaluated for potential development of vaccine products [5], [22]. The fact that the *Campylobacter* is commensal in poultry and that it shows high genetic and antigenic diversity among different strains make the development of an effective vaccine quite difficult. One of the promising vaccine candidates is an attenuated *Salmonella* strain carrying the *C. jejuni* CjaA antigen [22]. CjaA is a highly immunogenic protein that is well conserved among different *Campylobacter* serotypes and induces protective immune responses in chickens [22]. Cj0091 is also highly conserved among the 4 sequenced strains of *C. jejuni* (≥99% amino acid identity). This fact plus the findings from this study suggest that Cj0091 may be used as a potential vaccine candidate in the control of *C. jejuni* colonization in chickens. This possibility will be examined in future studies.

## Materials and Methods

### Bacterial strains, plasmids, and culture conditions

The various bacterial strains, mutants, and plasmids used in this study are listed in Table 1. *Campylobacter* strains were routinely grown in Mueller-Hinton (MH) broth (Difco, Detroit, MI) or agar at 42°C with an atmosphere of 5% O_2,_ 10% CO_2_, and 85% N_2_. *Escherichia coli* strains were grown is Luria-Bertani (LB) broth (Difco) or agar at 37°C with shaking at 250 rpm. Media were supplemented with ampicillin (Amp) (100 µg/ml), kanamycin (Km) (50 µg/ml), or chrolamphenicol (Cm) (4 µg/ml for *Campylobacter*, 20 µg/ml for *E. coli*) as needed.

### PCR

All primers used for PCR are listed in Table 2. PCR amplification was performed in a volume of 100 µl containing 200 µM of each deoxynucleoside triphosphate, 200 nM primers, 2.5 mM MgSO_4_, 100 ng of template DNA and 5 U of *Taq* DNA polymerase (Promega, Madison, WI) or *pfu Turbo* DNA polymerase (Stratagene, La Jolla, CA). Cycling conditions varied depending on the estimated melting temperatures of the primers and the expected size of the products. PCR products were purified with a QIAquick PCR purification kit (QIAGEN, Valencia, CA). For reverse transcriptase PCR (RT-PCR), total RNA was isolated from *C. jejuni* NCTC 11168 using the RNeasy minikit (QIAGEN). Isolated total RNAs were treated with RNase-free DNase (QIAGEN) to remove contaminating genomic DNA, and this was followed by quantification and qualification of the RNA using a NanoDrop microscale spectrophotometer (NanoDrop Technologies, Wilmington, DE). RT-PCR was conducted using the SuperScript™ III One-Step RT-PCR system with Platinum® *Taq* DNA Polymerase (Invitrogen, Carlsbad, CA). Cycling conditions for the RT-PCR were as follows: synthesis of cDNA at 50°C for 30 min; denaturation at 94°C for 2 min, followed by 40 cycles of 94°C for 15 s, 55°C for 30 s and 72°C for 2 min 30 s; and a final extension at 72°C for 10 min. The negative control was a RT-PCR mixture with the *Taq* polymerase (Promega) without the RT step, while the positive control was a reaction with genomic DNA as the template.

### Production of rCmeR

**F**ull-length recombinant CmeR was produced in *E. coli* as described previously [26], [27] with some modifications. Specifically, the two cysteine residues in CmeR (C69 and C166) were replaced by serine using site-directed mutagenesis to increase the stability of purified CmeR. The mutagenesis was performed using the QuikChange II site-directed mutagenesis kit (Stratagene). Complimentary primers CmeRC1-F (5′-GAAATTTTAGATGACATAAGTAAAAAACACTTTCATC) and CmeRC1-R (5′-GATGAAAGTGTTTTTTACTTATGTCATCTAAAATTTC-3′) were used to generate the C69S mutation, while primers CmeRC2-F (5′-CTTGCTGTTCTTTTTAGCACTATGTTAAAAGAACC-3′) and CmeRC2-R (5′-GGTTCTTTTAACATAGTGCTAAAAAGAACAGCAAG-3′) were used to generate the C166S mutation. The reaction was performed in a 50 µl mixture containing 5 µl of 10 × reaction buffer, 50 ng of purified pQE11168CmeR plasmid (*cmeR* cloned into pQE30) [27], 1 µl of each of the 10 µM primers (CmeRC1-F, CmeRC1-R, CmeRC2-F, and CmeRC2-R), 1 µl of dNTP mix, 1 µl of PfuUltra HF DNA polymerase (2.5 U/µl) and ddH_2_O. The temperature cycling used for synthesis and amplification started with 1 cycle at 95°C for 30 s and then followed by 18 cycles (95°C for 30 s, 55°C for 1 min, and 68°C for 4 min). The reaction was then cooled on ice for 2 min. In order to digest the parental DNA, 1 µl of the *Dpn* I restriction enzyme (10 U/µl) was added directly to the amplification reaction, which was then gently mixed and incubated at 37°C for 1 hour. Then, the *Dpn* I-treated DNA was transformed into competent *E. coli* JM109 cells. Positive colonies were selected on LB Agar plate containing ampicillin (100 µg/ml). The mutated plasmids were isolated and the desired mutations were confirmed by DNA sequencing. The rCmeR carrying the C69S and C166S changes were produced, purified and used for DNA binding assay as described previously [26].

### Deletional mutation of *cj0089*

An isogenic *cj0089* mutant was constructed by deletional mutagenesis in *C. jejuni* strain NCTC 11168. To construct the *cj0089* mutant, primers cj0089-F5 and cj0089-R5 (Table 2) were used to amplify the 1915-bp fragment containing the entire open reading frame (ORF) of *cj0089* (1362 bp) and its flanking sequences from *C. jejuni* strain NCTC 11168 chromosomal DNA. The PCR product was digested with *Sph*I and *Sac*I and cloned into pUC19 (Invitrogen) to form pUC19-89. Inverse PCR was performed using *pfu Turbo* DNA polymerase (Stratagene) on pUC19-89 using primers cj89-U2 and cj89-L2 (Table 2), which resulted in a 460-bp deletion within *cj0089*. A kanamycin-resistance (Kan^R^) cassette, amplified from pMW10 [51] by *pfu Turbo* DNA polymerase (Stratagene), was inserted into the amplified product to obtain pUC19-89K. The suicide vector, pUC19-89K, was introduced into *C. jejuni* NCTC 11168 by electroporation. Transformants were selected on MH agar containing Km (50 µg/ml). Disruption of *cj0089* by deletion of the partial ORF and insertion of the Kan^R^ gene with the correct orientation was confirmed by PCR, and the *cj0089* mutant was named Cj89^−^.

### Insertional mutation of *cj0091*

An isogenic *cj0091* mutant was constructed by insertional mutagenesis in *C. jejuni* NCTC 11168. The 1724-bp fragment containing the entire ORF of *cj0091* (624 bp) and its flanking sequences was amplified using primers cj0091-F2 and cj0091-R2 (Table 2) from *C. jejuni* NCTC 11168 chromosomal DNA. The PCR product was digested with *Sac*I and *Xba*I and cloned into pUC19, forming pUC19-91. pUC19-91 was digested with *Cla*I followed by Klenow treatment (Takara, Shiga, Japan) to form blunt ends. The Kan^R^ cassette, amplified from pMW10 by *pfu Turbo* DNA polymerase (Stratagene), was inserted into the *Cla*I-digested and Klenow-treated pUC19-91 to obtain pUC19-91K. The suicide vector, pUC19-91K, was introduced into *C. jejuni* NCTC 11168 by electroporation. Transformants were selected on MH agar containing 50 µg of Km per ml. PCR was performed to confirm the disruption of *cj0091* by the Kan^R^ gene with the right orientation, and the *cj0091* mutant was named Cj91^−^.

### Complementation of Cj91^−^

To construct the complement of Cj91^−^, primers 91comp-F2 and 91comp-R2 (Table 2) were first used to amplify the 744-bp fragment containing the entire ORF of *cj0091* (624 bp) and its flanking sequences from *C. jejuni* strain NCTC 11168 chromosomal DNA. The PCR product was digested with *Sac*I and *Sac*II and cloned into pRY112 [52], containing the promoter region of *cmeABC* to form pRY112-Comp91. Primers 91comp-F3 and 91comp-R2 were used to amplify the fragment containing the promoter region of *cmeABC* and entire ORF of Cj0091 from pRY112-comp91. The amplified product was digested with *Sac*I and *Xba*I and cloned into pUOA18 [53] to form pUOA18-Comp91. The vector, pUOA18-Comp91, was introduced into Cj91^−^ by conjugation. Transformants were selected on MH agar containing 50 µg of Km per ml and 4 µg of Cm per ml. PCR and immunoblotting were performed to confirm the complementation of Cj91^−^, and the complement of Cj91^−^ was named Comp91.

### Expression and purification of recombinant Cj0089 and Cj0091

6 x histidine (His)-tagged recombinant Cj0089 and Cj0091 (rCj0089 and rCj0091, respectively) were produced in *E. coli* using the pQE-30 vector of the QIAexpress System (QIAGEN). A 1323-bp sequence (not including the N-terminal lipoprotein signal peptide sequence) of *cj0089* (1362 bp) was amplified using primers cj0089F and cj0089R (Table 2). A 567-bp fragment (without the N-terminal lipoprotein signal peptide sequence) of *cj0091* (624 bp) was also amplified using primers cj0091-F1 and cj0091-R1 (Table 2). The amplified products containing restriction sites at the 5′-ends (Table 2) were purified and digested with appropriate restriction enzymes and separately ligated into pQE-30 vectors. The predicted lipoprotein signal peptides of Cj0089 and Cj0091 were not included in the recombinant products. Thus, the recombinant products represented the mature proteins in *C. jejuni*. Cloning, expression and purification of rCj0089 and rCj0091 were performed using the procedures provided by the QIAexpress system. The purified proteins were washed with 1 x PBS (containing 10 mM benzamidine) to remove imidazole and were concentrated using Ultracel®YM-30 (for Cj0089) and Ultracel®YM-10 (for Cj0091) Centrifugal Filter Units (Millipore, Billerica, MA).

### Production of rabbit antisera

A polyclonal rabbit antiserum against rCj0089 was generated in our laboratory. A New Zealand white rabbit was immunized three times with rCj0089 at a 2-week interval between the injections. Each immunization used 100 µg of rCj0089 emulsified in incomplete Freund's adjuvant. The animal was sacrificed 21days after the last injection to collect the antisera against rCj0089. Polyclonal antiserum against rCj0091 was generated in two rabbits by Pacific Immunology Corp (San Diego, CA, USA). The antisera were stored at −80°C for further use.

### Electrophoretic mobility shift assays (EMSA)

To determine whether CmeR directly binds to the operator region of *cj0089*, EMSA, as described by Alekshun et al. [54], was performed with slight modifications. The 179-bp promoter region of *cj0089* was amplified using primers Pcj89-F1 and Pcj89-R1 (Table 2). The amplified product was then labeled at the 3′ end with digoxigenin-11-ddUTP (DIG-11-ddUTP) using the DIG Oligonucleotide 3′-End Labeling kit (Roche Molecular Biomedicals, Indianapolis, IN). The DIG-11-ddUTP-labeled DNA (0.02 pmol) was incubated with different amounts of rCmeR (0, 20, and 40 ng) in 20 µl binding buffer containing 20 mM HEPES (pH 7.6), 1 mM EDTA, 10 mM (NH_4_)_2_SO_4_, 5 mM dithopthreitol, 0.2% Tween-20, 30 mM KCl, and 50 ng of poly(dI-dC). The reaction mixtures were incubated at room temperature for 30 min, followed by electrophoresis on a nondenaturing 6% (w/v) polyacrylamide gel in a 0.5 x TBE buffer (44.5 mM Tris, 44.5 mM boric acid, 1 mM EDTA, pH 8.0) at 200 V for 45 min. The DNA was transferred from the gel to a positively charged nylon membrane using a vacuum blotter. Alkaline phosphatase-conjugated anti-DIG antibody and the chemiluminescent substrate CDP-star (Roche Molecular Biochemicals) were sequentially applied to the membrane. The chemiluminescence signals on the blots were captured using a digital imaging system (ChemiImager™5500 from Alpha Innotech, San Leandro, CA). The promoter DNA of *cmeABC* and the intragenic fragment of *cmeB* were used as the positive and negative controls, respectively, as described in a previous study [27].

### Preparation of cell fractions

The cell fractions of cytoplasm, periplasm, inner membrane, and outer membrane were obtained using the method described by Leon-Kempid et al. [55], with slight modifications. In total, 500 ml of an overnight culture of *C. jejuni* strain NCTC 11168 were harvested by centrifugation at 5000 x *g* for 30 min, and the cell pellet was resuspended in 20 ml of the ST buffer (20% w/v sucrose, 30 mM Tris-HCl, pH 8.0) at room temperature. EDTA was added to the cell suspension to a final concentration of 1 mM and the suspension was incubated for 10 min at room temperature with shaking. The cells were then centrifuged at 8000 x *g* for 10 min at room temperature to collect the cell pellet. The supernatant was discarded. The pellet was resuspended and stirred in ice-cold 10 mM Tris-HCl (pH 7.5) at 4°C for 10 min followed by centrifugation at 15,000 x *g* at 4°C for 15 min. The supernatant was concentrated by 10% TCA and kept as the periplasmic fraction. The pellet was again resuspended in 5 ml of ice-cold 10 mM Tris-HCl (pH 7.5), followed by sonication (Virsonic, an SP Industries Company, Warminster, PA) by three bursts of 30 s at 6 µm amplitude and centrifugation at 13,000 x *g* at 4°C for 15 min. The pellet was discarded. The supernatant was ultracentrifuged at 100,000 x *g* at 4°C for 1 hour. The supernatant was then transferred to a new tube and again centrifuged at 100,000 x *g* at 4°C for 1 hour. The supernatant was concentrated by 10% TCA, corresponding to the cytoplasmic fraction. The pellet collected was the total membrane fraction. The isolation of inner and outer membrane was performed using the method of Carlone et al. [56]. The pellet of membrane fraction was washed three times with ice-cold 10 mM Tris-HCl (pH 7.5) and resuspended in 0.2 ml of 10 mM HEPES buffer (pH 7.4). An equal volume of sarkosol buffer (2% sodium N-lauroylsarkosine; SIGMA) was added and incubated at room temperature for 60 min, during which the solution was mixed occasionally. The inner membrane proteins were solubilized at this step. The mixture was centrifuged at 15,600 x *g* for 30 min at 4°C. The supernatant was kept as the soluble inner membrane fraction. The pellet was washed with 0.5 ml of 10 mM HEPES buffer followed by resuspension in 200 µl of 10 mM HEPES buffer. This fraction was the outer membrane fraction. The fractions were analyzed by SDS-PAGE and immunoblotting.

### Adhesion assays

Adhesion assays were performed according to the method described by Jin et al. [20], with some modifications. INT 407 cells (ATCC CCL-6) were grown in Minimum Essential Medium (MEM) (GIBCO, Invitrogen) containing 10% fetal bovine serum (FBS), 100 µg/ml streptomycin, and 100 units/ml penicillin G (GIBCO, Invitrogen) at 37°C under the atmosphere of 5% CO_2_. Once the INT 407 cells became confluent, the monolayers were trypsinized and approximately 1×10^5^ cells/well in MEM with 10% FBS without antibiotics were applied to each well of a 24-well tissue culture plate, which was incubated at 37°C for 18 h under the atmosphere of 5% CO_2_. The monolayers were washed twice with 1 x PBS. Overnight fresh cultures of NCTC 11168, Cj89^−^, Cj91^−^, and Comp91 were added to wells containing monolayers in 1 ml of MEM with 10% FBS at an INT 407 cells-to-bacteria ratio of 1∶100, and incubated at 37°C under the atmosphere of 5% CO_2_ for 3 h. The monolayers were washed five times with 1 x PBS. The washed monolayers were then lysed with 0.2 ml of 1% (w/v) Triton X-100 in PBS for 15 min. The *Campylobacter* cells were enumerated by plate counting on MH agar plates. All assays were repeated three times and were done in quadruplicate.

### Motility assay

Bacterial strains were grown overnight and their OD_600_ was adjusted to ca. 0.5. A single stab inoculation was made in the center of the soft agar (0.4%; wt/vol) and the plates were incubated at 42°C for 48 hours. The swarming diameters of the strains were measured at the widest portion of the swarm halo.

### Colonization in chicken

To examine whether the mutations of *cj0089* and *cj0091* affected *C. jejuni* colonization in chickens, 3-day-old commercial broilers (Ross x Cobb), purchased from a commercial hatchery, were inoculated via oral gavage with approximately 10^7^ CFU of NCTC 11168, Cj89^−^, Cj91^−^, or Comp91 (each group consists of 15 birds). At necropsy, cecal samples were collected from 5 birds from each group at 3, 6, and 10 days post inoculation (DPI), homogenized, serially diluted in 1 x PBS, and plated on MH plates containing *Campylobacter*-specific selective agents and growth supplements (SR084E and SR117E; Oxoid). After two-day incubation at 42°C under microaerophilic conditions, the *Campylobacter* colonies were counted.

### Ethics Statement

All animals were handled in strict accordance with the recommendations in the Guide for the Care and Use of Laboratory Animals of the National Institutes of Health. The animal use protocol was approved by the Institutional Animal Care and Use Committee of Iowa State University (A3236-01). All efforts were made to minimize suffering of animals.
